# The Year-Book of the Nose, Throat and Ear

**Published:** 1903-03

**Authors:** 


THE YEAR-BOOK OF THE NOSE, THROAT AND EAR.—The Nose and Throat, edited by G. P. Head, M. D., Professor of Laryngology and Rhinology in the Post-Graduate Medical School, Chicago. The Ear, edited by Albert H. Andrews, M. D., Professor of Otology, Post-Graduate Medical School, Chicago. The Year-Book Publishers, 100 State Street, Chicago.

A full review of the year's literature on the ear, throat and nose appears in this volume. Those interested in these special subjects will find this work very satisfactory. T. J. B.

